# Editorial: Molecular characterization of thyroid lesions in the era of “next generation” techniques: volume II

**DOI:** 10.3389/fendo.2024.1460239

**Published:** 2024-07-29

**Authors:** Umberto Malapelle, Claudio Bellevicine, Alex Friedlaender, Alessia Ciarrocchi, Dario de Biase

**Affiliations:** ^1^ Department of Public Health, University Federico II of Naples, Naples, Italy; ^2^ Oncology Service, Clinique Générale Beaulieu, Geneva, Switzerland; ^3^ Laboratory of Translational Research, Azienda Unità Sanitaria Locale-IRCCS di Reggio Emilia, Reggio Emilia, Italy; ^4^ Department of Pharmacy and Biotechnology (FaBiT), University of Bologna, Bologna, Italy; ^5^ Solid Tumor Molecular Pathology Laboratory, IRCCS Azienda Ospedaliero-Universitaria di Bologna, Bologna, Italy

**Keywords:** thyroid tumors, molecular pathology, preoperative evaluation, biological markers, thyroid associated disease

Thyroid cancer research has advanced dramatically in recent years. This Research Topic features a mix of cutting-edge studies on various aspects of thyroid cancer, ranging from molecular profiling to innovative prediction models for recurrence, providing fresh insights and prospective routes for improved patient care. Advanced diagnostic strategies may help to better classify thyroid tumor entities and predict their biological behavior, as prognosis or response to therapy. Thyroid tumors could be further classified using different risks and predictive factors. The second volume of our Research Topic on the molecular characterization of thyroid lesions comes at a pivotal time in thyroid pathology. This Research Topic delves into the intricate molecular mechanisms underlying thyroid disorders, emphasizing advancements brought by next-generation techniques.

The increasing prevalence of thyroid nodules, identified mostly by ultrasonography, contrasts strongly with the consistent death rates of thyroid malignancies. This disparity emphasizes the need for accurate molecular and genetic characterization to distinguish between indolent and aggressive thyroid tumors. The papers in this Research Topic look at novel diagnostic, prognostic, and predictive tools that use enhanced imaging, histology, and molecular analysis to improve our knowledge and management of thyroid illnesses.

Metabolomic investigations on thyroid cancer have been done to define pathological subgroups, predict distant metastases, and identify biomarkers for prognosis and diagnosis (1, 2).

Zhou et al. investigated the metabolic effects of thyroidectomy in individuals with papillary thyroid carcinoma (PTC). The untargeted metabolomic analysis reveals considerable dysregulation in lipid metabolism following surgery, emphasizing the importance of thorough post-operative metabolic surveillance. The authors found that molecular pathways such as cysteine and methionine metabolism and aminoacyl-tRNA biosynthesis are associated with PTC post-operative thyroidectomy complications, such as fatigue and insomnia. Interestingly, differential metabolic influences of hemi- and total thyroidectomy were observed: in fact, the authors described that, even if a great portion of these metabolites were identical, some metabolites were different between surgery and 1-month-after surgery in patients who underwent thyroidectomy versus hemithyroidectomy. These findings not only expand our understanding of the systemic consequences of thyroidectomy but also open the way for more effective patient treatment measures targeted at improving long-term quality of life.

Given the high frequency of thyroid nodules with a relatively small percentage of malignant changes (1-5%), distinguishing between benign and malignant lesions is extremely important in reducing the number of needless surgical procedures. Biomarkers include thyroglobulin (TG), anti-thyroglobulin antibodies (ATGAbs), and calcitonin (CT) are frequently employed in preoperative diagnosis and postoperative follow-up of patients with thyroid nodules, differentiated thyroid carcinomas, and medullary thyroid cancer (3, 4).

Samardzic et al. investigated the role of nitric oxide (NO), thyroglobulin (TG), and calcitonin (CT) in serum and fine-needle aspiration biopsy (FNAB) washouts in relation to thyroid nodule cytology findings, according to the Bethesda system for thyroid cytopathology (5), with the aim of improved and more accurate identification of the thyroid nodules. Their study, involving 86 subjects, reveals that TG levels in FNAB washouts (TGw) correlate positively with cytology findings classified by the Bethesda system, suggesting higher TGw levels indicate higher malignant potential. The study further explores the levels of NO in FNAB washouts (NOw), noting that higher NOw levels correspond to more suspicious ultrasound findings, although these levels did not significantly correlate with the Bethesda categories.

The findings underscore the potential of TGw and NOw as predictive markers for malignancy in thyroid nodules. Specifically, TGw levels could serve as a valuable preoperative indicator, aiding clinicians in stratifying patients based on their risk and potentially reducing unnecessary surgeries for benign conditions. Moreover, the study suggests that incorporating NOw levels, alongside traditional ultrasound and cytology assessments, could enhance the accuracy of malignancy risk stratification, particularly in nodules with indeterminate cytology.

Regarding the importance of distinguishing between benign and malignant lesions in pre-operative material, Yang et al. created a prediction model for positive FNAB results in C-TIRADS 4 thyroid nodules, demonstrating the utility of a prediction model (nomogram) based on clinical and ultrasound predictors. Yang et al. have presented a comprehensive prediction model intended to improve the diagnosis accuracy of FNAB in C-TIRADS 4 thyroid nodules. The authors analyzed about 550 individuals to identify five independent predictors of positive FNAB: age lower than 45 years, low echo, microcalcifications, aspect ratio (>1), and irregular morphology. This nomogram, proven using complex statistical studies such as ROC curves and decision curve analysis, has an AUC of 0.793, indicating high discriminating capacity and clinical usefulness within the threshold probability range of 14% to 91%. The obtained nomogram represents a useful tool for clinicians since it allows for customized risk assessment for patients with C-TIRADS 4 thyroid nodules. By combining ultrasound findings and patient demographics, our model assists in making educated judgments about the need for FNAB, potentially decreasing unneeded intrusive procedures. The study emphasizes the importance of specific ultrasound features, such as low echo and microcalcifications, in predicting malignancy, reinforcing their role in the comprehensive evaluation of thyroid nodules.

The 2015 American Thyroid Association (ATA) recommendations recommend using molecular markers to predict the recurrence of differentiated thyroid carcinomas, such as PTC (6). The 2022 World Health Organization Classification (WHO) of Thyroid Neoplasms introduces molecular subtypes of PTC as a new classification (7).

In a pivotal study by Li et al., a predictive model for recurrence in papillary thyroid carcinoma (PTC) was developed based on a retrospective cohort study of almost 1000 cases. The model incorporates variables such as age, extranodal extension, metastatic lymph node ratio, and treatment history, demonstrating high predictive accuracy. Key risk factors identified included age equal to or higher than 55 years, extranodal extension, metastatic lymph node ratio >0.5, and non-initial treatment. Their model demonstrated high predictive accuracy, with an AUC of 0.819 in the training group and 0.818 in the validation group. This model provides clinicians with a valuable tool to predict the risk of PTC recurrence and tailor follow-up and management plans accordingly. The identification of these risk factors fills a critical gap in the prognostic tools for the non-initial treatment population.

The introduction of multigene molecular panels and next-generation sequencing technologies has been thoroughly investigated in the hopes of improving diagnostic accuracy among indeterminate thyroid nodules (8–11). The study by Craig et al. introduces a novel machine-learning algorithm (HighLifeR) applied to RNA-Seq data from The Cancer Genome Atlas (TCGA) (12). This approach has identified 82 genes closely associated with recurrence in PTC, enabling the classification of tumors into three distinct molecular subtypes. With their study, Craig et al. devised a molecular classifier that outperforms the ATA Risk Stratification system in predicting the likelihood of recurrence. Using this machine learning algorithm PTC nodules are stratified into three different groups characterized by a specific prognostic signature and having significant potential to direct clinical decisions: (i) Ras-like Type 1 PTCs, enriched with mutations in *NRAS* and *HRAS* and characterized by very different inflammatory microenvironment, compared to Type 2 and 3 PTCs; (ii) Type 2 PTCs, harboring *BRAF* V600E mutation in over half and with the lower expression levels of immunoregulatory genes; (iii) Type 3 PTCs, comprised the majority of *TERT* promoter mutations and characterized by an abundant immune cell infiltrate and higher expression of immunoregulatory molecules, and that may also be amenable to immune checkpoint inhibitors. The genomic classifier developed by Craig et al. can be applied preoperatively, potentially guiding the extent of surgery and the need for adjuvant therapies. This personalized approach could minimize overtreatment in patients with low-risk tumors while ensuring aggressive management for those with high-risk profiles. The integration of genomic data with clinical features marks a significant step toward precision medicine in thyroid cancer.

In their comprehensive study, Du et al. provided a detailed genetic landscape of thyroid cancer in Chinese patients, involving 458 cases across multiple subtypes of thyroid malignancies most of them with PTC (95.6%), followed by ATC (2.2%), FTC (1.3%), and PDTC (0.9%). Utilizing a 1,021-gene panel for next-generation sequencing, the authors identified an average of 2.1 alterations per patient, and *BRAF* was the most commonly (76.0%) mutated gene. Other frequently altered genes were the *RAS* gene family, *TP53*, *PI3KCA*, and *PTEN*. *TERT* promoter alterations were observed in 6.3% of cases and were usually accompanied by oncogenic driver mutations. Intriguingly, the number of somatic mutations and TMB were dramatically greater in *TERT* mutated cases than in *TERT* WT ones. Moreover, three novel gene fusions have been documented, including two in-frame gene fusions between *RET* and other genes (*GRIPAP1* and *GRAMD3*) and one gene fusion between *BRAF* and *PAK1*. Their findings underscore the heterogeneity of thyroid cancer and its clinical implications, particularly highlighting the association of somatic mutations with adverse clinical characteristics. This extensive molecular profiling offers a critical reference point for tailoring personalized therapeutic strategies in thyroid cancer treatment.

Hashimoto’s thyroiditis (HT) is an autoimmune condition in which the immune system attacks and destroys the thyroid glands. Although the etiology of HT is known to be complicated, involving genetic, environmental, and epigenetic variables, the specific underlying processes are unknown. Furthermore, HT is linked to an increased risk of several malignant cancers, including papillary thyroid carcinoma (PTC) (13–15). Liu et al. investigate the shared molecular mechanisms between PTC and HT. By employing bioinformatics methods on datasets from the Gene Expression Omnibus (GEO), the authors identify differentially expressed genes (DEGs) and key shared genes between PTC and HT. The authors queried HT (GSE138198) and PTC-related datasets (GSE33630) to screen the expression of key genes shared by HT and PTC. This study emphasizes the interconnected nature of chronic inflammation and carcinogenesis in the thyroid gland. The identification of key genes and pathways offers new avenues for targeted therapies and improved diagnostic strategies. By understanding the molecular basis of HT and PTC, clinicians can better predict disease progression and tailor treatments accordingly. Moreover, this research underscores the importance of next-generation sequencing and bioinformatics in uncovering complex disease mechanisms. The combination of massive datasets and powerful analysis tools opens the door to more customized medical approaches in thyroid pathology.

The contributions to this Research Topic highlight the dynamic and complex character of thyroid cancer research. Each study provides unique insights into the genetic, molecular, and clinical aspects of thyroid cancer, expanding our understanding of the illness and opening the path for more effective and individualized treatment options. As we continue to explore the complexity of thyroid cancer, these findings will provide the groundwork for future research and clinical innovation.

## Author contributions

UM: Writing – review & editing, Writing – original draft. CB: Writing – review & editing, Writing – original draft. AF: Writing – review & editing, Writing – original draft. AC: Writing – review & editing, Writing – original draft. DdB: Writing – review & editing, Writing – original draft.
